# Bottom‐Gate Approach for All Basic Logic Gates Implementation by a Single‐Type IGZO‐Based MOS Transistor with Reduced Footprint

**DOI:** 10.1002/advs.201901224

**Published:** 2020-01-24

**Authors:** Shaocheng Qi, Joao Cunha, Tian‐Long Guo, Peiqin Chen, Remo Proietti Zaccaria, Mingzhi Dai

**Affiliations:** ^1^ School of Materials Science and Engineering Shanghai University Shanghai 200444 China; ^2^ Ningbo Institute of Materials Technology and Engineering Chinese Academy of Sciences Ningbo 315201 P. R. China; ^3^ Cixi Institute of Biomedical Engineering Ningbo Institute of Materials Technology and Engineering Chinese Academy of Sciences Ningbo 315201 China; ^4^ University of Chinese Academy of Sciences Beijing 100049 China; ^5^ Istituto Italiano di Tecnologia via Morego 30 16163 Genoa Italy

**Keywords:** amorphous oxide semiconductors (AOSs), In–Ga–Zn–O (IGZO), integrated circuits, logic gates, metal‐oxide‐semiconductor field‐effect transistors (MOSFETs)

## Abstract

Logic functions are the key backbone in electronic circuits for computing applications. Complementary metal‐oxide‐semiconductor (CMOS) logic gates, with both n‐type and p‐type channel transistors, have been to date the dominant building blocks of logic circuitry as they carry obvious advantages over other technologies. Important physical limits are however starting to arise, as the transistor‐processing technology has begun to meet scaling‐down difficulties. To address this issue, there is the crucial need for a next‐generation electronics era based on new concepts and designs. In this respect, a single‐type channel multigate MOS transistor (SMG‐MOS) is introduced holding the two important aspects of processing adaptability and low static dissipation of CMOS. Furthermore, the SMG‐MOS approach strongly reduces the footprint down to 40% or even less area needed for current CMOS logic function in the same processing technology node. Logic NAND, NOT, AND, NOR, and OR gates, which typically require a large number of CMOS transistors, can be realized by a single SMG‐MOS transistor. Two functional examples of SMG‐MOS are reported here with their analysis based both on simulations and experiments. The results strongly suggest that SMG‐MOS can represent a facile approach to scale down complex integrated circuits, enabling design flexibility and production rates ramp‐up.

## Graphical abstract

With the purpose of addressing the complementary metal‐oxide‐semiconductor (CMOS) scaling down issue, a single‐type channel multigate metal‐oxide‐semiconductor (MOS) transistor (SMG‐MOS) capable of reducing the CMOS footprint down to 40% or even less is here presented. Importantly, the SMG‐MOS still holds the two important aspects of processing adaptability and low static dissipation of CMOS.

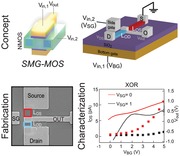

## 1 Introduction

Logic function gates are the basic and fundamental elements enabling data processing in electronic integrated circuits (IC).^1^, ^2^, ^3^, ^4^, ^5^, ^6^ Complementary metal‐oxide‐semiconductor (CMOS) transistors are one of the dominating components in the modern logic gate family as they are characterized by a remarkable adaptability to modern foundry lines and low leakage current, which ensure high fabrication reliability/repeatability and low power dissipation, respectively.^4^, ^5^

**Figure 1:**
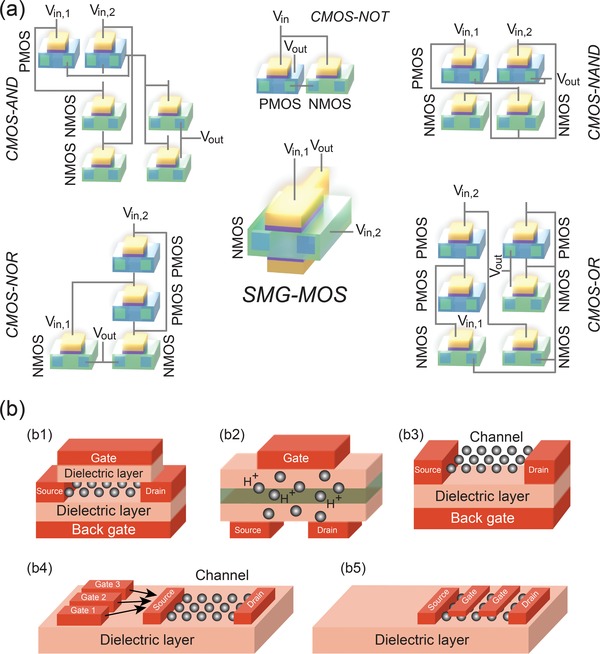
Comparison of CMOS logic circuits and SMG‐MOS circuit. a) The SMG‐MOS circuit shows a footprint about 40% of a CMOS circuit performing the same logic function. These schematics follow the foundry line (top gate approach). b) Schematic comparison of bottom gate‐like solutions employing external‐channel (i.e., not directly connected to the channel) gates (b1–b4) with our internal‐channel (i.e., directly connected to the channel) gate approach (b5). In particular, (b1) is a representation of the device concept from ref. 36, (b2) from refs. 37, 38, 39, (b3) from refs. 40, 41, and (b4) from refs. 42, 43, 44, 45. Finally, (b5) shows the configuration proposed in the present work. Importantly, this configuration employs a reduced number of material layers than (b1–b3) and a lower surface coverage than (b4).

For CMOS logic gates, a minimum number of two metal‐oxide‐semiconductor (MOS) field‐effect transistors (FETs) are required to achieve the simplest Boolean logic gate, the logic NOT (**Figure** **1**). Specifically, an n‐type channel MOS (NMOS) field‐effect transistor (FET) and a p‐type channel MOS (PMOS) FET are used. CMOS logic gates are built by employing two different semiconductor materials, resulting in material and processing complexity. Furthermore, to accomplish two‐input Boolean logic gates with CMOS requires more than two transistors. According to the well‐known Moore's law, the number of transistors per square inch on an IC should be doubled every two years, which is equivalent to scaling down the dimension of each transistor by half. Until recent years, this steady technological advancement allowed for an equally steady improvement of computers performance, especially in terms of computational speed. A scaled down circuit can indeed enable a faster logic data transporting and processing. However, lithographic techniques constraining quantum effects and limited dopant placement capabilities have currently begun to interfere with Moore's law.

To extend the benefits of the Moore's law, highly complex and expensive innovative fabrication processes as well as new logical schemes are required.^1^, ^2^, ^3^, ^4^, ^5^, ^6^, ^7^, ^8^ In this regard, several different alternative logic gates have been investigated,^9^, ^10^, ^11^, ^12^, ^13^, ^14^, ^15^, ^16^, ^17^, ^18^, ^19^, ^20^, ^21^, ^22^, ^23^, ^24^, ^25^, ^26^, ^27^, ^28^, ^29^, ^30^, ^31^, ^32^, ^33^ such as nanotube gates,^9^, ^10^, ^11^, ^12^, ^13^ 2D materials logic gates,^15^, ^16^, ^17^, ^18^ quantum logic gates,^19^, ^20^, ^21^, ^22^, ^23^ and biocircuits.^26^, ^27^, ^28^ For instance, quantum logic gates are scalable using existing silicon technologies, but they demand very low working temperatures.^22^, ^23^, ^24^, ^25^ Biocircuits employ biological elements, which are difficult to control and are highly sensitive to working conditions including temperature, with the drawback of not being suitable for nowadays foundry processing lines since they require very different fabrication equipment facilities very different from the existing semiconductor foundry product lines.^26^, ^27^ Several of these alternatives share this drawback failing the important requirement of relying on the present processing schemes as many of the proposed designs are undeveloped trials which are not immediately implementable.^34^, ^35^ Rather than updating the processing, materials and working environments, the manipulations of the transistor design provides an easier and cheaper approach as it can be implemented via an easy and simple modification of existing manufacturing tools and recipes.

Here, we design, fabricate, characterize, and simulate a single‐type channel multigate MOS transistor (SMG‐MOS) meeting the requirements of scaling down capability, low static power dissipation, and compatibility with nowadays foundry lines, where no extra instruments and processing are required. A single SMG‐MOS transistor can work as different basic Boolean logic gates, such as NAND, NOT, AND, NOR, and OR, which otherwise would require a large number of CMOS transistors (Figure 1). The different logic functions are obtained by producing different electric field profiles along the channel, which can be controlled by adjusting both the positioning of the transistor electrodes contacts along the channel and the applied voltages. Indeed, the SMG‐MOS design utilizes a single‐type channel structure with an output electrode added directly inside the channel, fabricated through a single‐type channel transistor processing. The simplicity and functionality of the present SMG‐MOS design results in a facile approach for scaling down integrated circuits. Indeed, it significantly decreases the number of transistors typically required for a logic circuit hence reducing the 2D footprint, power consumption, and cost while increasing processing speed and ensuring mass production capability. Importantly, the proposed architecture is profoundly different from standard double/multigate solutions both in terms of geometrical design and working principle. Specifically, in terms of geometrical design, in our proposed architecture a gate is added inside the channel (i.e., directly connected to it) whereas in standard double‐ or multigate structures a gate is added outside the channel (i.e., there is no direct connection to the channel). Furthermore, in terms of working mechanism, while standard double/multi‐gate solutions are based on a not‐localized electric field generation in the overall channel, where the side gates are used to control the channel, in our proposed design we use only specific portions of the channel to operate the output (i.e., the portion of the channel at the drain side is used to assign status 1 to the output, and the portion of the channel at the source side to assign status 0 to the output). These are fundamental differences leading to an important footprint reduction for our SMG‐MOS design. For clarity, a visual representation of the illustrated concept is shown in Figure 1b.

## 2 Results and Discussion

### 2.1 SMG‐MOS Fabrication and Logical Description

**Figure 2:**
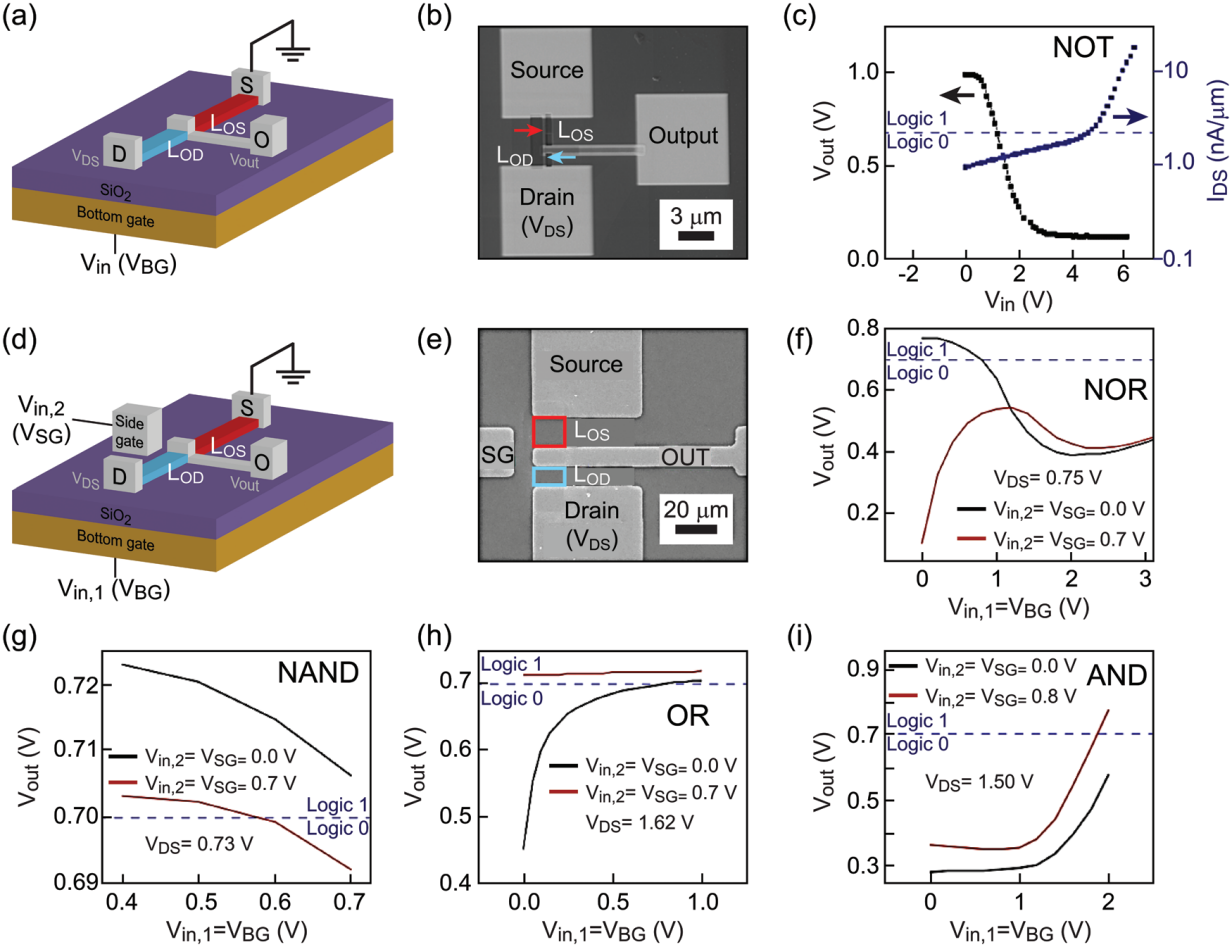
SMG‐MOS transistor configuration. a) 3D illustration of a SMG‐MOS logic NOT, with the bottom gate voltage *V*_BG_ (*V*_in_), the source (S), the drain (D), the semiconductor channel (light blue and red color) and the output (O). The figure highlights the different length between *L*_OS_ and *L*_OD_. b) SEM image of a SMG‐MOS logic NOT (channel width = 200 nm, channel thickness = 10 nm). *L*_OS_ is the channel section comprised between the output and the source, and *L*_OD_ is the channel section comprised between the output and the drain. c) Plots of the output voltage (*V*_out_) and channel current (*I*_DS_) as a function of *V*_in_. d) 3D illustration of a two‐input SMG‐MOS logic gate capable of addressing any other Boolean gates besides NOT. SG denotes a side gate providing the side gate voltage (*V*_SG_), i.e., the second input *V*_in,2_. e) SEM top‐view of the two‐input logic gate (channel thickness = 30 nm). f–i) Plots of *V*_out_ as a function of *V*_in,1_ with *V*_in,2_ taken as parameter for the logic gates NOR (f), NAND (g), OR (h), AND (i).

In this study, we implement a research lab line approach to realize the SMG‐MOS concept, with the employment of a bottom gate as shown in **Figure** **2** in order to simplify the fabrication conditions. This approach is slightly different from the SMG‐MOS shown in Figure 1 that instead depicts a foundry line approach. As shown in Figure 2a, the transistor is formed by drain (D), source (S), and output (O) electrodes with the semiconductor channel highlighted in blue and red colors. These elements are located on the top of a 100 nm thick SiO_2_ layer. The layer electrically insulates the elements and an n‐type silicon bottom gate (sheet resistance << 0.005 Ω cm^2^). The semiconductor channel, with a thickness between 20 and 50 nm, is an n‐type In–Ga–Zn–O (IGZO). IGZO is an amorphous oxide semiconductor (AOS) composite widely investigated and utilized in the thin film transistors (TFTs) field especially for displays and sensors.^46^, ^47^, ^48^, ^51^, ^53^ The electrodes are composed of a 60 nm thick Ni/Au alloy. The overall structure was fabricated following standard top‐down fabrication techniques. The details of the fabrication process can be found in the Experimental Section as well as in previous publications.^46^, ^47^, ^48^, ^49^, ^50^, ^51^, ^52^, ^53^ Figure 2b,e shows the SEM images of the fabricated SMG‐MOS devices, where the former highlights the smallest semiconductor channel with 200 nm width we could fabricate, and the latter represents the typical structure employed in our characterization.

We defined *V*_GS_ as the gate‐to‐source voltage, *V*_out_ as the output voltage, *V*_DS_ as the drain‐to‐source voltage and *I*_DS_ as the channel current measured from the drain terminal. Here, *V*_GS_ can be further specified as bottom gate voltage *V*_BG_ or side gate voltage *V*_SG_. The threshold voltage of *V*_BG_ is defined as *V*_Th_. A simplified nomenclature associates *V*_in_ to *V*_GS_. If two inputs are required (depending on the considered logic gate), *V*_in,1_ and *V*_in,2_ will be considered corresponding to *V*_BG_ and *V*_SG_, respectively, as also revealed in Figure 1. Regarding operation conditions, for both *V*_in_ and *V*_out_, we define the logic condition 1 with a voltage value no less than 0.7 V.^14^ In turn, a voltage value lower than 0.7 V corresponds to logic 0.

Bearing this in mind, when a standard CMOS logic NOT circuit is considered (Figure 1), if *V*_in_ corresponding to logic 0 is applied, *V*_out_ is “pulled up” to logic 1 by the PMOS transistor. On the other hand, if *V*_in_ of logic 1 is applied, *V*_out_ is “pulled down” to logic 0 by the NMOS transistor.^4^ Differently from the CMOS logic design, the SMG‐MOS assigns the output close to the drain, to “pull up” the output by the drain and “pull down” the output by the source (Figure 2b). By doing so, the output‐to‐drain part of the channel, named *L*_OD_, could be turned on before the entire channel. Hence, different output values and thus logic gate functions could be implemented with careful positioning of the output. In the SMG‐MOS logic NOT (Figure 2a), when *V*_in_ is a logic 0, the *L*_OD_ is turned on. This will enable *V*_out_ to be pulled up by *V*_DS_ to a voltage value more than 0.7 V, meaning *V*_out_ logic 1.^21^ On the contrary, when *V*_in_ is a logic 1, the output‐to‐source section *L*_OS_ is turned on, which connects *V*_out_ to the grounded source and shifts output to logic 0. Figure 2c shows the dependence of *V*_out_ and the channel current *I*_DS_ on *V*_in_ producing the function of a logic NOT (inverter). A detailed description of the overall mechanism is provided by Figure S1 (Supporting Information) and related text. Based on the same working principle, the SMG‐MOS logic NOT was also capable of driving the second stage in a two‐stage NOTs, producing similar results to CMOS NOT stages (Figure S2, Supporting Information).

For the remaining Boolean logic gates (Figure 1a), two inputs are required (*V*_in,1_ and *V*_in,2_). In order to fulfill this requirement, we add a side gate (SG) to the SMG‐MOS NOT component shown in Figure 2d. A representative top‐view SEM image is presented in Figure 2e. All basic Boolean logic gates can be implemented. In this respect, the SMG‐MOS *V*_out_ as a function of the two inputs *V*_in,1_ and *V*_in,2_ for the four basic logic gates is shown in Figure 2f (NOR), Figure 2g (NAND), Figure 2h (OR), and Figure 2i (AND). Furthermore, *V*_SG_ is shown to enable the control of *V*_Th,_ which defines the “activate” condition of the channel (Figure S3, Supporting Information). In Figure 2a, number of different voltage conditions are shown, each of them associated with a specific logic gate. Generally speaking, for a relatively small drain and gate voltages, the AND logic gate can be implemented, whereas for a relatively high drain and gate voltages, the OR logic gate can be implemented.

Finally and for comparison purposes, the representative standard Boolean NMOS logic gates are shown schematically in Figure S4a (Supporting Information) while the corresponding SMG‐MOS design is illustrated in Figure S4b (Supporting Information) with the associated truth tables in Figure S4c (Supporting Information). These results suggest that a single SMG‐MOS can implement all basic Boolean operations, while performance improvement can be achieved by advanced lithography and proper material selection.

### 2.2 Mechanism Investigation

**Figure 3:**
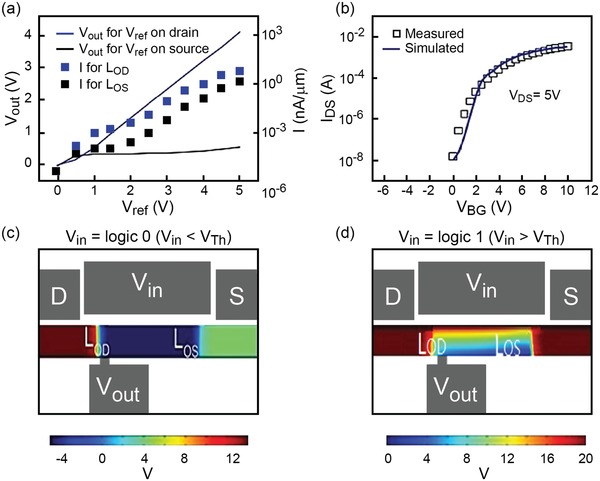
Mechanism of SMG‐MOS logic NOT gate function. a) *V*_out_ versus reference voltage *V*_ref_ applied either on the drain (blue line) or source (black line). *V*_out_ is lower than *V*_ref_ when *V*_ref_ is applied to the source, which confirms the isolation between the output and the source when the input *V*_in_ = logic 0 (*V*_in_ < *V*_Th_). The dotted data show that the channel current is lower than the leakage current of 100 nA µm^−1^. b) Comparison between simulated (line) and experimental (scatter points) of *I*_DS_ vs *V*_BG_. c) Schematic representation of simulated electric potential along the channel at *V*_in_ = logic 0, with only *L*_OD_ being activated. d) Schematic diagram of simulated potential at *V*_in_ = logic 1, with the activation of the entire channel.

The mechanism underneath our design is revealed in a quantitative way through the electrical measurements shown in **Figure** **3**.^42^ In SMG‐MOS logic NOT, a reference voltage *V*_ref_ was applied to either the source or drain while *V*_out_ was measured to confirm the proper connection among the electrodes (Figure 3a). The increase of *V*_out_ (blue line) with *V*_ref_ applied to the drain confirms there is an electrical connection between the drain, implying *L*_OD_ is turned on. On the other hand, when *V*_ref_ is applied to the source, *V*_out_ (black line) remains constant, confirming there is no connection between the source and output electrodes and demonstrating *L*_OS_ is turned off. The presence/absence of an electrical connection describes a logic NOT gate function. In this regard, TCAD simulations have been performed to determine the contribution to the *V*_out_ values coming from *L*_OD_ and *L*_OS_.^52^, ^53^ Simulation results (simulation parameters in Figure S5, Supporting Information) are found to fit well the experimental results in Figure 3b. A schematic illustration with the electric potential distribution along the channel as obtained from TCAD simulations is shown in Figure 3c,d. In particular, the contour plot in Figure 3c shows high electrical potential exclusively concentrated on the drain side when *V*_in_ = logic 0 (*V*_in_ < *V*_Th_), which confirms previous discussion. As seen in Figure 3c, when the transistor is turned off then almost the entire channel is without carriers or associated appreciable electric field. In fact, only the electric field at the drain side, due to the high drain voltage stress, is strong enough to turn on that specific portion of the channel (drain side). In particular, if the output electrode is close enough to the drain, then only *L*_OD_ is turned on meaning a high localized electric field in the channel at the drain side. On the other hand, the remaining portion of the transistor channel remains in off condition, meaning a low electric field in the rest of the channel and hence low current. The overall situation results in the channel undergoing on/off states for *L*_OD_ and *L*_OS_, respectively. Additionally, the electric potential extents along the whole channel when *V*_in_ = logic 1 (*V*_in_ > *V*_Th_) which suggests that the electrical connection between the output and the source induces *V*_out_ = logic 0 (Figure 3d). This localized distribution of potential confirms the working mechanism of SMG‐MOS logic NOT.

**Figure 4:**
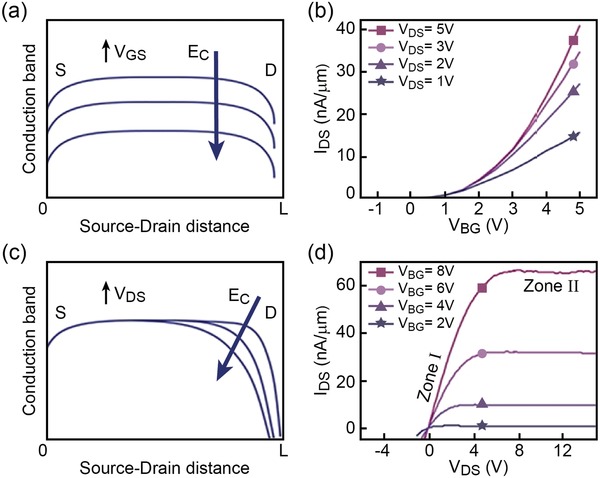
Conduction band explanation of general SMG‐MOS logic gates. a) Schematic diagram showing that *E*_c_ along the entire channel decreases as the gate voltage *V*_GS_ increases. b) *I*_DS_ versus *V*_GS_ (here *V*_BG_) plot suggesting the *V*_GS_ impact on the channel at varying *V*_DS_. c) Schematic diagram showing that only the part of *E*_c_ near the drain is bended down when *V*_DS_ is increased. d) *I*_DS_ versus *V*_DS_ plot at varying of *V*_GS_ (here *V*_BG_). In (a,c) the *L* is the distance between source and drain.

In general, SMG‐MOS logic operations can be explained using the band diagram formulation.^4^ In equilibrium conditions, there is no current flow along the channel given the existence of an energy barrier between the Fermi level *E*_F_ and the conduction band *E*_c_. However, when *E*_c_ is pulled down below *E*_F_ due to an applied voltage bias, the conduction band carriers turn into mobile channel carriers.^4^ In this respect, either *V*_GS_ (side gate or bottom gate voltage) or *V*_DS_ can bend *E*_c_ and thus adjust *V*_out_ through the modification of the channel conductivity. **Figure** **4**a shows *E*_c_ along the whole channel and how it is bent in a uniform way by applying a *V*_GS_ bias. The resulting channel current *I*_DS_ is depicted in Figure 4b. Different from *V*_GS_, the quantity *V*_DS_ can control *E*_c_ only on the drain edge, as shown schematically in Figure 4c. Therefore, for high values of *V*_DS_, only *L*_OD_ is turned on,^54^ leading to a short circuit between the output and drain and pulling up *V*_out_ to logic 1 with *V*_in_ = logic 0. This situation is suitable for the implementations of logic NOT, logic NAND, or logic NOR, which require *V*_out_ to be logic 1 when *V*_in_ = logic 0.

From Figure 4d, a typical output curve of an n‐type transistor is shown. Two distinct behaviors can be retrieved when the channel is on: a linear behavior (zone I) followed by a flat behavior (zone II). The zone I corresponds to the electric field distributed along the channel in an average way so that the entire channel behaves like a resistor. In this case, the channel resistance and thus *V*_DS_ is linearly dependent on the channel length. The zone II, on the other hand, describes a situation where the channel at the drain edge is in saturation condition. The *L*_OD_ connection leads to *V*_out_ = logic 1 whereas the remaining part of the channel *L*_OS_, not being in saturation condition, leads to *V*_out_ = logic 0.^4^ Therefore, *V*_out_ = logic 1 generally could occur in three cases: i) when *L*_OD_ is on but *L*_OS_ is off, hence *L*_OD_ pulls *V*_out_ up close to *V*_DS_; ii) when the transistor is working in zone II, with the output‐channel connection located within the saturation region on the drain edge; iii) when the transistor is working in zone I so that the channel is turned on, working as a resistor, and the output‐channel connection is located in a position to hold a high enough *V*_DS_ value.

### 2.3 Proof of Concept Demonstration

**Figure 5:**
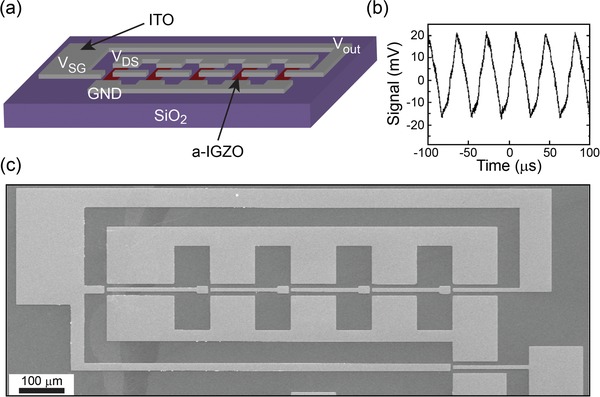
RO circuit. a) Schematic illustration of a five‐stage SMG‐MOS NOT ring oscillator. b) Output voltage as a function of time for the five‐stage SMG‐MOS RO at *V*_DS_ = 2 V. c) SEM image of a five‐stage SMG‐MOS RO (top view).

In order to demonstrate the suitability of the SMG‐MOS logic gates in the fabrication of complex logic circuits, we have implemented our concept to two different kinds of circuits. The first implementation example is a ring oscillator (RO) circuit. The RO plays an important role in microprocessors since it provides the clock signals as a timer to define when each functional unit starts and stops working. A typical RO can be formed by combining different logic NOTs. Here, a five‐stage SMG‐MOS logic NOT is fabricated with one stage connecting the following stage hence forming the RO circuit as schematically shown in **Figure** **5**a (see Experimental Section). The labels *V*_DS_, *V*_out,_ and GND correspond to the supplied voltage, the output voltage, and the source ground voltage, respectively. Figure 5b demonstrates the functionality of the RO. When *V*_DS_ = 2 V, the output voltage oscillates as a function of time. Figure 5c illustrates a SEM image of such device (top view), which was fabricated via a standard lithography procedure suggesting that it could be easily adaptable to foundry lines for mass production. Importantly, already at the lab scale, we could reduce the number of necessary transistors of half with respect to the standard CMOS approach. At present, the SMG‐MOS RO showed an oscillation frequency limited only by the intrinsic property of IGZO and the parasitic resistance due to the device dimension.^55^, ^56^, ^57^ The mobility of the IGZO transistors here is around 1 cm^2^ V^−1^ s^−1^, which is much lower than poly‐Si (≈100 cm^2^ V^−1^ s^−1^).^46^ We believe that the oscillation frequency would be enhanced with a proper choice of channel materials and by improving the structure dimensions through more advanced foundry lithography. Even though the illustrated result sounds very promising, it must be highlighted the low resulting voltage output. This is ascribed to the use of the side gate as input instead of the bottom gate, the latter one a solution which could probably improve the performance of the RO due to the stronger effect of the bottom gate on the SMG‐MGO output.

The second implementation example consists of a half adder circuit by making use of AND and XOR logic functions. A half adder circuit can realize the addition of two single binary digits as input 1 and input 2 by producing two outputs, the sum (S or output 1) and the carry (C or output 2). A half adder is important for electronics because two half adders compose a full adder which is a fundamental component in the arithmetic logic. A half adder structure can be realized through the combination of logic AND and XOR gates, i.e., through the employment of two SMG‐MOS. A half adder is an adder not taking into consideration the carry from the lower order. A half adder has two inputs and two outputs, the latter formed by the sum value and the carry. When either the input 1 or input 2 is 1, the sum value is 1 with the carry returning 0. When both inputs are 1, the sum is 0 and the carry is 1. The complete truth table for the half adder circuit is shown in **Table** **1**.

**Table 1:** Half adder truth table

Input 1	Input 2	Sum value	Carry
0	0	0	0
0	1	1	1
1	0	1	1
1	1	0	0

**Figure 6:**
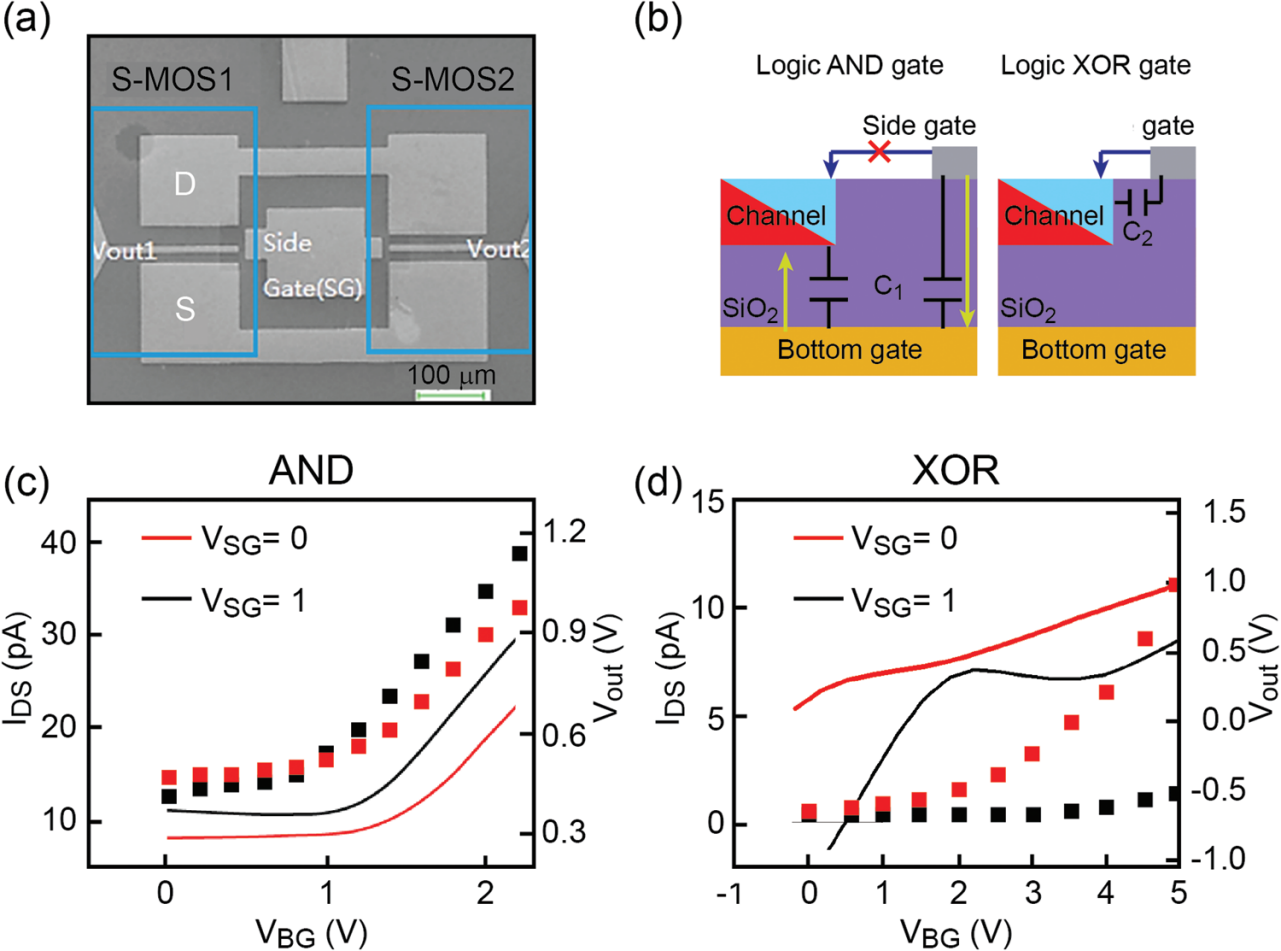
Half‐adder circuit. a) SEM image of a half adder with logic XOR (SMG‐MOS1) and logic AND (SMG‐MOS2). b) Cross‐section schematic circuit diagrams of a SMG‐MOS looked from the drain side into the channel, with the arrows showing two routes through which *V*_SG_ controls the channel: i) Yellow arrow: route through capacitors *C*_1_; ii) Blue arrow: route through capacitor *C*_2_. c) Transfer curves for logic AND, showing the transfer current *I*_DS_ (squares) shifting to the left by increasing *V*_SG_. The continuous lines represent *V*_out_. The red color stands for *V*_SG_ = logic 0, black colors for *V*_SG_ = logic 1). d) Transfer curve for logic XOR. The curve associated to I_DS_ shifts to the right by increasing *V*_SG_. Here *V*_BG_ = *V*_in1_ and *V*_SG_ = *V*_in2_.

As shown in **Figure** **6**a, a half adder circuit was implemented by employing a SMG‐MOS logic AND and a logic XOR. The XOR logic gate standard implementation requires the use of several transistors or, alternatively, of a single SMG‐MOS. In Figure 6a, the two SMG‐MOS logic gates highlighted in blue square blocks share the same drain, source, side gate, and bottom gate. These two SMG‐MOS logic gates have the same channel length but two different distances from the side gate to the channel, referred as *L*_SC_. In particular, SMG‐MOS1 has shorter *L*_SC_ than SMG‐MOS2, resulting in the implementation of XOR logic and AND logic, respectively.

The working mechanism is as follows. As shown in the cross‐section of the circuit in Figure 6b, there are generally two different routes for *V*_SG_ to control the channel: i) control of the bottom of the channel through capacitor *C*_1_ and bottom gate, as shown by the yellow arrow route in the left side of Figure 6b; ii) control of the top or side of the channel through capacitor *C*_2_, as shown by the blue arrow route in the right side of Figure 6b. In particular, for the logic AND gate, *L*_SC_ is long enough that the route represented by the blue arrow is not accessible. In this case, *V*_SG_ controls the channel through the route represented by the yellow arrow, pointing toward the bottom gate voltage. This is consistent with the experimental results depicted in Figure 6c, showing the transfer curve shifting to the left by increasing *V*_SG_. For the logic XOR gate shown in Figure 6b, *L*_SC_ is instead small enough to turn on the blue arrow route. *V*_SG_ can control the top of the channel, now in the opposite direction of the bottom gate *V*_BG_. In this case, when *V*_SG_ increases, a higher *V*_BG_ is required to turn on the channel for the same *I*_DS_. This is consistent with the experiments in Figure 6d showing a slight shift towards the right (the electric potential values are shown in Figure S6, Supporting Information).

Based on this information, the logic XOR can be implemented by adopting the following procedure. To start *V*_SG_ = logic 0, therefore *L*_OD_ is inactive when *V*_BG_ corresponds to logic 0. When instead *V*_BG_ shifts from logic 0 to logic 1, the channel is activated and *V*_out_ = *I*_DS_∙*R*_OS_ shifts from logic 0 to logic 1 (where *R*_OS_ is the channel resistance from the output to source). The next situation considers instead *V*_SG_ = logic 1, situation where *L*_OD_ is turned on at *V*_BG_ = logic 0 which results in *V*_out_ = logic 1. Finally, when also *V*_BG_ = logic 1 then a relatively smaller *I*_DS_ is generated leading to *V*_out_ = *I*_DS_∙*R*_OS_ < logic 1 condition (i.e., logic 0). By this reasoning the XOR table truth is formed.

We have seen how the SMG‐MOS solution carries a number of advantages toward standard CMOS technology. As for the footprint and thus the area, by using fewer transistors, SMG‐MOS structures are smaller than the corresponding CMOS logic gates in the same technology node. In particular, for the 0.13 µm technology node, the SMG‐MOS logic NOT footprint results to be around 40% of the standard CMOS‐NOT (with the surface area of ≈1.84 µm^2^ with effective reduction in the required number of fabrication steps would be achieved. Finally, an evident advantage would result from the instrumentation point of view as the present processing lines, 14 nm CMOS technology node, could allow the realization of SMG‐MOS circuits with footprint equivalent to 10 nm CMOS technology node. This possibility sounds especially appealing considering that updating the standard 14 nm to a 10 nm technology node is estimated to require one billion dollars for processing and 0.3 billion for designing.

The SMG‐MOS architecture presents an advantage over CMOS also in terms of low power dissipation, protecting circuits from heating, which degrades performance. In general, the static power dissipation of a single transistor is proportional to *I*_DS_∙*V*_DD_, where *V*_DD_ is the working voltage. SMG‐MOS transistors work in the subthreshold region around 0.7 V (Figure 2c,f–i), so that *I*_DS_ can be kept lower than the leakage current limit (100 nA µm^−1^) and *V*_DD_ lower than the working voltage limit (≈1.5 V). The limits of 100 nA µm^−1^ and 1.5 V are required by the International Technology Roadmap for Semiconductors (ITRS), followed by the foundries across the world^5^ (see section Electrical Properties Measurement for further details). Finally, as for the mass production, SMG‐MOS is processed via standard lithography and thus relatively easy to adapt into foundry lines with high repeatability and reliability.

## 3 Conclusions

The advancement of semiconductor technology toward faster and more efficient data processing will soon require innovative technological solutions as the physical limitations of materials is getting closer. In this respect, the conventional scaling down methods show several limitations. For example, CMOS logic gate circuits employing high number of transistors are hard to scale down, while bench‐top innovations for advanced miniaturized components cannot always be implemented into common previous product lines quickly or efficiently. Therefore, there is a need for designs, which can be fabricated by employing existing foundry CMOS technologies. The SMG‐MOS design presented herein has demonstrated the efficiency and flexibility of executing multiple logic gate functions. SMG‐MOS logic gates carry the important advantage in reducing footprint and costs (in time, materials, and processing steps) required to achieve the same function of CMOS logic gates. In addition, the SMG‐MOS logic gates have a lower static power consumption with respect to CMOS logic gates. As demonstration, we have implemented a ring oscillator circuit by using SMG‐MOS logic NOTs, with the result of reducing the transistor number by half when compared to a standard ring oscillator circuit realized with CMOS logic NOTs. As a further example, SMG‐MOS can also be used as data calculator circuits for data processing in IC such as half adders. Finally, no particular requirement needs to be added to conventional semiconductor processing for SMG‐MOS, which suggests that SMG‐MOS can be adopted by the existing semiconductor foundry lines. Owing to the simplicity of the design, its low power consumption and low cost, the SMG‐MOS concept could represent a new possible way to scale down electronics circuits.

## 4 Experimental Section

*Materials*: A layer of SiO_2_ with thickness 100 ± 20 nm acting as insulator layer was grown on the surface of n‐type silicon. The employed Si wafer was characterized by a 2 in. diameter, crystal orientation <100>, resistance less than 0.005 µ cm^2^ with thickness of 400–500 µm. Substrates were purchased from Suzhou Yancai Micro‐nano Scientech Corp. (Taipei, Taiwan, China). Indium–gallium–zinc oxide (IGZO) and Ni/Au (Beijing Founder Star Science and Technology Co., Ltd., China) were deposited and patterned in sequence on the SiO_2_/Si wafer. Ni/Au was used as electrodes due to the good at adhesive property of Ni and good electric conductivity of Au.

*Fabrication*: A 30 nm thick IGZO film was sputtered using a Manual Radio Frequency Magnetron Sputterer at 100 W from Sky Technology Development (Shenyang, China) with a 0.9 Pa working pressure (Ar:O_2_ = 14 sccm:3 sccm) at 50 °C. The resulting channel width and length were ≈15–100 and 30–120 µm, respectively (see Figure 2e). The channel was submitted to a 20 h 200 °C annealing process. Afterwards, Ni/Au metal electrodes of ≈12–60 nm thickness were deposited by MUE‐ECO electron‐beam‐evaporation using an E‐beam evaporator from ULVAC (Redwood City, CA, USA). The pressure was 1.8 × 10^−3^ Pa, and deposition rate no less than 0.06 nm s^−1^. Finally, the electrodes for the output (O), source (S), drain (D), and side gates (SG) were patterned by lithography with a resolution of 1 µm. In terms of best resolution, by employing a Focus Ion Beam the smallest achievable length between the drain and output was 1 µm with 200 nm width.

*Electrical Measurements*: The *I*–*V* characteristics were measured using a semiconductor parameter analyzer (Keithley 4200), where the source voltage was set to ground (i.e., 0 V). For *I*_DS_–*V*_GS_ measurements, i.e., the typical transfer curves, the drain voltage *V*_DS_ was set in the range 1.0–1.5 V. The low leakage, hence the low static power dissipation, is explained in more details as follows: i) *I*_DS_ at input = 0 and 1 is much lower than 100 nA µm^−1^, as shown in Figure 2c. 100 nA µm^−1^ is the leakage current limit required by the IC International Technology Roadmap for Semiconductors (ITRS) after scaling down for the present technology node.^5^ ii) The drain voltage used in the SMG‐MOS is in the range of 1–2 V, which is the typical working voltage according to ITRS.^5^ iii) The static power dissipation for a single device is the product of channel current and drain voltage (*I*_DS_∙*V*_DD_). Therefore, given the three aforementioned considerations, the static power consumption of a SMG‐MOS logic NOT will be comparable to or even lower than the power consumption required by a traditional CMOS NOT.

The Ni/Au metal electrode‐IGZO channel contact was also investigated.^58^, ^59^, ^60^ As shown in Figure S7 (Supporting Information), both the role of the IGZO thickness and the resistance of the metal‐IGZO contact were investigated. The experiments suggest an ohmic contact between metal and IGZO, as highlighted by the linear relationship between current and voltage. More details can be found in the Supporting Information.

Another important aspect that was taken into consideration is the effect of the side gate on the transfer curve of the SMG‐MOS as might result in an important parameter for controlling the transistor electrical properties. Indeed, as shown in Figure S8 (Supporting Information), the side gate was found to be capable of significantly increase the drain current.

Furthermore, because the static power dissipation is proportional to the number of transistors *n* in the logic gate, the power dissipation in other SMG‐MOS logic gate designs could be smaller than that of CMOS logic gate for the same technology nodes, because of fewer transistors. The logic NOT function is repeatable in our samples for more than 10 devices. The OR, AND, NAND, NOR functions are also repeatable. The RO functions are measured in more than five samples in one wafer.

Finally, stability measurements to address the sensitivity of IGZO from the surrounding ambient were performed. In particular, as shown in Figure S9 (Supporting Information), a solution was introduced to minimize the effect of the ambient temperature.

*Simulations*: The single‐transistor circuit structure was simulated by TCAD as follows: First, the mesh was defined by splitting the channel into 50 equally sized slices of 1 µm each (total length 50 µm), and the spatial structure of device was formed. A conductive layer as bottom gate was used as a substrate, onto which silicon oxide was considered. A 40 nm thick n‐type channel was then added on top, with doping concentration of ≈10^18^ cm^−3^. The side gates, source, and drain contacts were then realized. Subsequently, the physical properties for semiconductor and dielectrics materials could be specified, including mobility (≈1–10 cm^2^ V s^−1^), subgap density states of holes and electrons (10^18^–10^21^ cm^−3^ eV^−1^), energy bandgap at room temperature (≈3.0 eV) and the Shockley–Read–Hall recombination time for holes and electrons (≈10^−8^ s). Afterward, models based on semiconductor device theories were defined,^3^, ^4^ by including the recombination model, tunneling model, the output‐to‐drain electric field linkage condition introduced by the special design, and the device degradation model based on hot carrier injection. Defects were also defined by employing the density of states model^50^, ^51^, ^52^ while the employed material parameters (Figure S5, Supporting Information) were taken from published data.^50^, ^52^, ^53^ Finally, the bias conditions including the voltage applied at drain, source and gate were defined, and the full Newton method was employed to numerically calculate the electrical properties of the illustrated devices.

## Conflict of Interest

The authors declare no conflict of interest.

## Supporting information

Supporting Information
