# Supplementary material for: Bottom‐Gate Approach for All Basic Logic Gates Implementation by a Single‐Type IGZO‐Based MOS Transistor with Reduced Footprint
Source: Adv Sci (Weinh). 2020 Jan 24;7(6):1901224. doi: 10.1002/advs.201901224 (PMC7080509; doi:10.1002/advs.201901224)
Supplement: Supplementary file 1 — Supporting Information [file ADVS-7-1901224-s001.pdf]

Copyright WILEY-VCH Verlag GmbH & Co. KGaA, 69469 Weinheim, Germany, 2019.

ADVANCED SCIENCE Open Access

Supporting Information

for *Adv. Sci.,* DOI: 10.1002/advs.201901224

Bottom-Gate Approach for All Basic Logic Gates Implementation by a Single-Type IGZO-Based MOS Transistor with Reduced Footprint

Shaocheng Qi, Joao Cunha, Tian-Long Guo, Peiqin Chen, Remo Proietti Zaccaria, and Mingzhi Dai**

SUPPORTING INFORMATION

Bottom-gate approach for all basic logic gates implementation by a single-type IGZO-based MOS transistor with reduced footprint.

Shaocheng Qi,[†,‡] *Joao Cunha,*[‡] *Tianlong Guo,*[‡] *Peiqin Chen,*[‡] *Remo Proietti Zaccaria,**[‡,§] *Mingzhi Dai**[‡]

[†] School of Materials Science and Engineering, Shanghai University, Shanghai, 200444, China

[‡] Ningbo Institute of Materials Technology and Engineering, Chinese Academy of Sciences, Ningbo 315201, P. R. China.

§ Istituto Italiano di Tecnologia, via Morego 30, 16163 Genoa, Italy

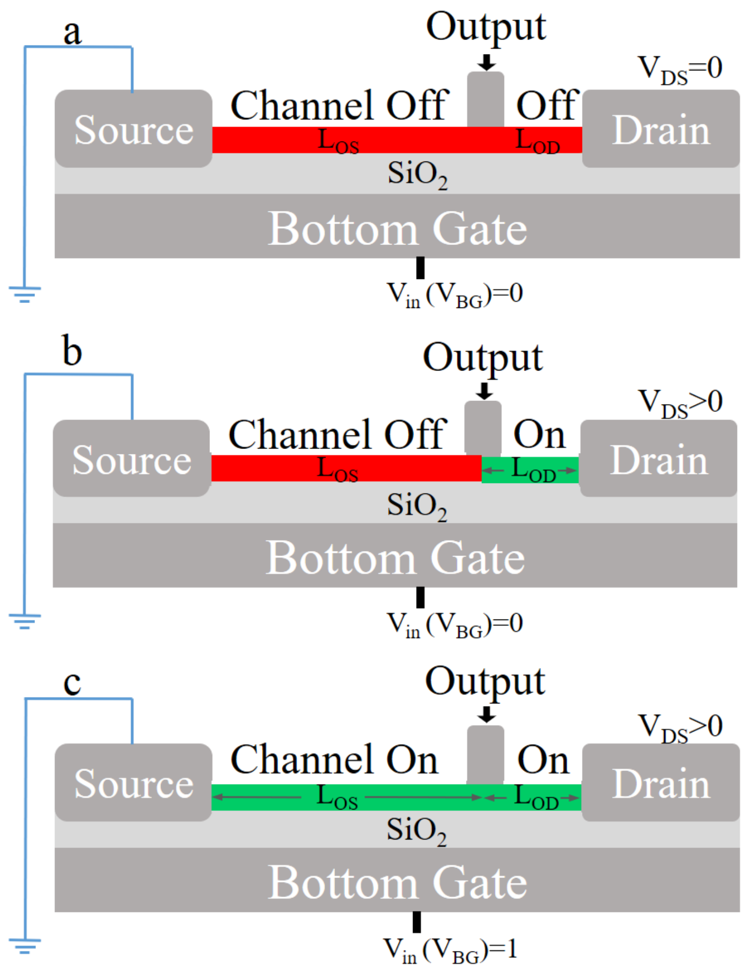

Figure S1. Working mechanism. Channel conductivity when (a) V_{in} and V_{DS} are both 0 V. (b) V_{in} is 0 V and V_{DS} is 1 logic state. (c) V_{in} and V_{DS} are both 1 logic state.

As we can see in the above figures, the output electrode (Output shown in Figure S1) positioned above the channel is located closer to the drain electrode than the source. By this choice we can divide the total channel into two parts, namely the output-to-source channel L_{OS} and the output-to-drain channel L_{OD}.

Furthermore, the source electrode is clearly grounded hence its voltage V_S is always 0 V, hence $V_{DS}= V_D-V_S= V_D$. The channel conductance is then controlled by the voltage applied at the

bottom gate (named V_{in} or V_{BG}) and by the drain electrode (V_{DS}). A non-conductive channel means the channel to be turned off, which can be made conductive (turned on) by V_{in} and V_{DS}.

Specifically, **Figure S1a** shows the situation when V_{in} and V_{DS} are both zero volt, i.e., V_{in}=0. In this case, the whole semiconductor channel is non-conductive, namely the channel is turned off. **Figure S1b** shows instead the situation when V_{in}= 0 V and V_{DS}>0 V. In this case, the portion of channel between the output electrode and the drain electrode (L_{OD}) is turned on due to a large enough drain-to-gate electric field, whereas the portion of channel between the output electrode and the source electrode (L_{OS}) remains non-conductive (i.e., the channel off). In this way, the output electrode could obtain a high voltage from the drain electrode. That is to say, a low input voltage (V_{in}= 0 V) causes a high output voltage (V_{out}=V_{DS}). Finally, **Figure S1c** shows the situation when V_{in}= 1 logic state and V_{DS}>0 V, situation corresponding to the whole channel turned on. In this configuration the channel can be seen as a resistor with the associated electric potential falling from the drain side to the source side (source is grounded and its electric potential V_S is zero volts). Or, from another perspective, the electrical potential of the output electrode is lowered by the grounded source electrode. That is to say, a high input voltage (V_{in}= 1 logic state) lowers the output voltage (V_{out}<V_{DS}).

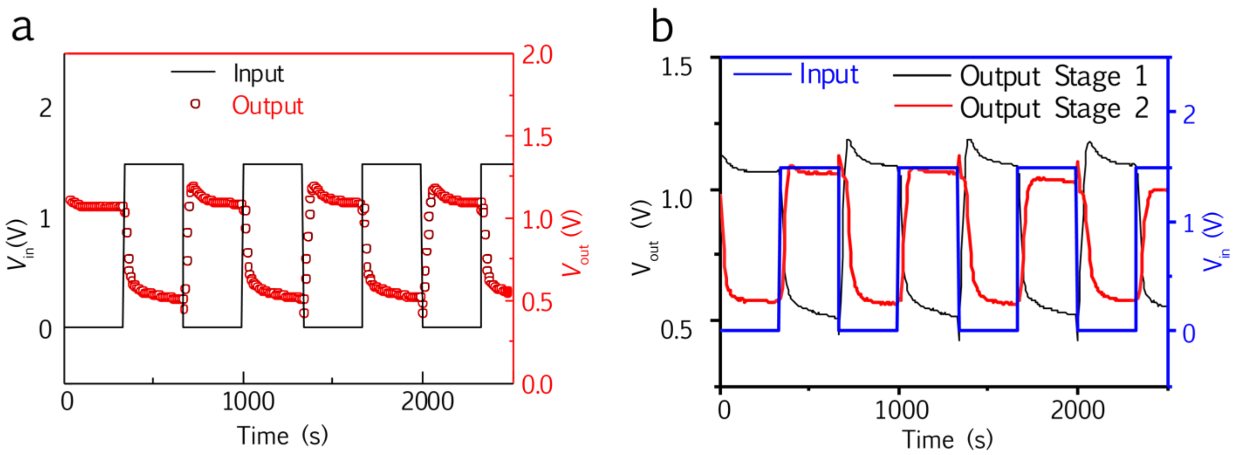

Figure S2. Additional measurements for SMG-MOS logic NOT function. (a) Output and input vs. time for a logic NOT gate, demonstrating circuit function repeatability. (b) Two-stage NOT measurements, demonstrating the ability to drive the next stage of logic in a complex circuit.

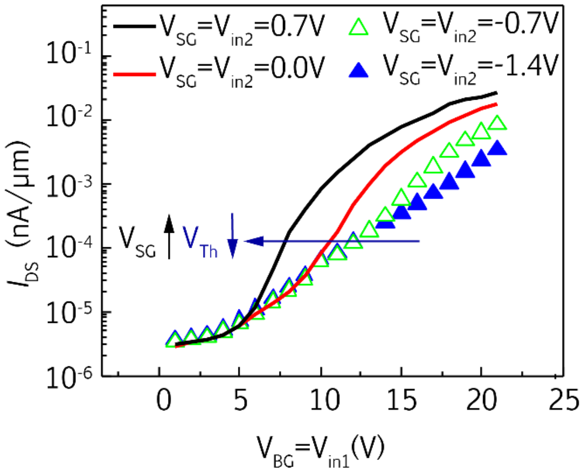

Figure S3. Effect of side gate on channel current. Bottom gate transfer curves of the transistor circuit by varying V_{SG}. The plot shows that V_{SG} can effectively modify the threshold voltage V_{Th}.

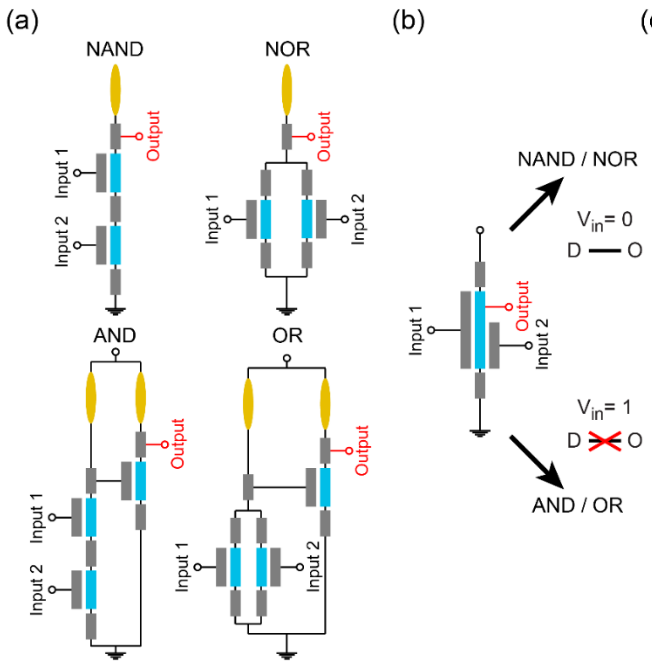

(c)

Logic NAND (V_{DS}= 0.73 V)

Input 1	Input 2	Output
0 (0.4 V)	0 (0.0 V)	1 (0.72 V)
1 (0.7 V)	0 (0.4 V)	1 (0.70 V)
0 (0.0 V)	1 (0.7 V)	1 (0.71 V)
1 (0.7 V)	1 (0.7 V)	0 (0.69 V)

Logic NOR (V_{DS}= 0.75 V)

Input 1	Input 2	Output
0 (0.0 V)	0 (0.0 V)	1 (0.77 V)
1 (1.0 V)	0 (0.0 V)	0 (0.11 V)
0 (0.0 V)	1 (1.2 V)	0 (0.53 V)
1 (1.0 V)	1 (1.2 V)	0 (0.55 V)

Logic AND (V_{DS}= 1.50 V)

Input 1	Input 2	Output
0 (0.0 V)	0 (0.0 V)	0 (0.28 V)
1 (2.2 V)	0 (0.0 V)	0 (0.68 V)
0 (0.0 V)	1 (0.8 V)	0 (0.37 V)
1 (2.2 V)	1 (0.8 V)	1 (0.77 V)

Logic OR (V_{DS}= 1.62 V)

Input 1	Input 2	Output
0 (0.0 V)	0 (0.0 V)	0 (0.10 V)
1 (0.7 V)	0 (0.0 V)	1 (0.71 V)
0 (0.0 V)	1 (0.8 V)	1 (0.70 V)
1 (0.7 V)	1 (0.7 V)	1 (0.72 V)

Figure S4. NMOS vs. SMG-MOS. (a) Configurations of NMOS transistors required to realize the logic gates NAND, NOR, AND and OR. (b) Similarly to (a) the SMG-MOS transistor is employed. If the connection between the drain and output is strong enough, either the logic NAND or the logic NOR are implemented (top); Logic gates AND/OR are also realized (bottom). (c) Truth tables for SMG-MOS logic NAND, NOR, AND and OR.

Key Parameters in TCAD Simulation	
E_g (eV)	3.0
Channel Dopant (cm^{-3})	$5x10^{17}$
μ_{FFF} ($cm^2/V\ s$)	1–10
N_c ($cm^{-3}eV^{-1}$)	$1x10^{20}$
N_v ($cm^{-3}eV^{-1}$)	$1x10^{20}$

Figure S5. TCAD simulation parameters. Key parameters for simulation which are in reasonable range of the previous references for IGZO transistors.[19]

Band diagram formulation.

With regard to **Figure 4d**, two distinct behaviours can be retrieved when the channel is on: a linear behaviour (zone I) followed by a flat behaviour (zone II). The zone I corresponds to the electric field distributed along the channel in an average way so that the entire channel behaves like a resistor. In this case the channel resistance and thus V_{DS} is linearly dependent on the channel length. The zone II, on the other hand, describes a situation where the channel at the drain edge is in saturation condition. The L_{OD} connection leads to V_{out}= logic 1 whereas the remaining part of the channel L_{OS}, not being in saturation condition, leads to V_{out}= logic 0.[4] Therefore, V_{out}= logic 1 generally could occur in three cases. i) When L_{OD} is on but L_{OS} is off, hence L_{OD} pulls V_{out} up close to V_{DS}; ii) When the transistor is working in zone II, with the output-channel connection located within the saturation region on the drain edge; iii) When the transistor is working in zone I so that the channel is turned on, working as a resistor, and the output-channel connection is located in a position to hold a high enough V_{DS} value.

Logic XOR (V_{DS}= 0.73 V)

Input 1	Input 2	Output
0 (0.0 V)	0 (0.0 V)	0 (0.28 V)
1 (2.2 V)	0 (0.0 V)	1 (0.74 V)
0 (0.0 V)	1 (0.7 V)	1 (-1.18 V)
1 (2.2 V)	1 (0.7 V)	0 (0.33 V)

Half adder (V_{DS}= 0.75 V)

Input 1	Input 2	Output 1	Output 2
0 (0.0 V)	0 (0.0 V)	0	0
1 (2.2 V)	0 (0.0 V)	0	1
0 (0.0 V)	1 (0.7 V)	0	1
1 (2.2 V)	1 (0.7 V)	1	0

Figure S6. Truth tables for SMG-MOS logic XOR and half adder. In reference to Fig. 6d, here Input 1 is V_{BG} and Input 2 is V_{SG}.

Figure S7 shows the IV plot highlighting the influence of IGZO channel thickness on the channel current and the metal/channel contact resistance. The motivation is the employment in our experiments of Ni/Au metal electrodes which may induce the formation of an n (IGZO)-p(NiO) junction. For this reason the interface layer needed to be investigated in order to verify any possible impact on the carriers transport. As shown by **Figure S7a**, to thinner IGZO films corresponds lower conductivity of the channel. Furthermore, the resistance of the n (IGZO)-Ni(Au) is depicted in **Figure S7b**. The linear relationship between voltage and current resulting from our experiments demonstrates an ohmic kind of contact, hence suggesting that no n (IGZO)-p(NiO) junction (diode) is formed.

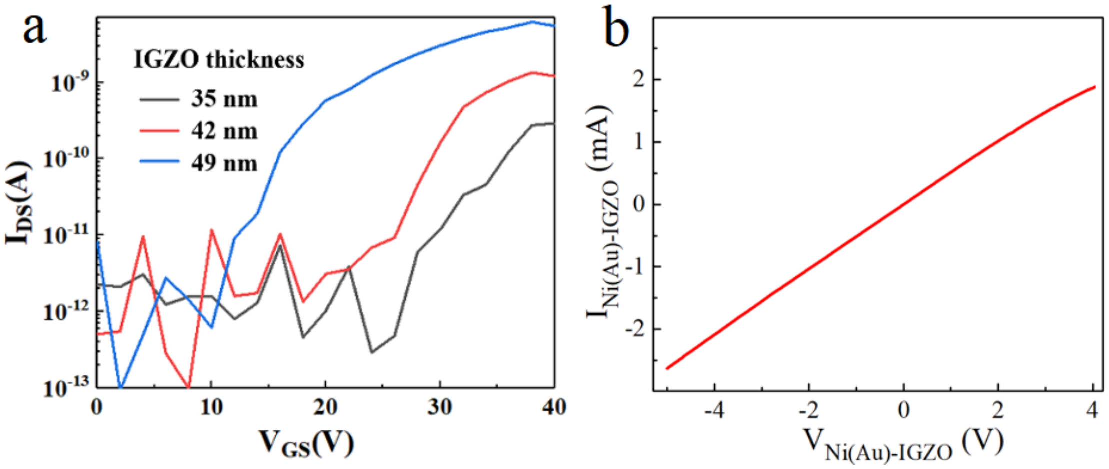

Figure S7. Metal-IGZO contact study. (a) Thickness dependence of the transfer curve. (b) Metal-IGZO channel resistance measurement, which suggests an ohmic type contact.

Figure S8 highlights the effect of the side gate used as input in a SMG-MOS. In particular, a side gate voltage increase determines a vertical shift towards higher currents of the IV curves, hence implying a noticeable influence on the logic gates electric property.

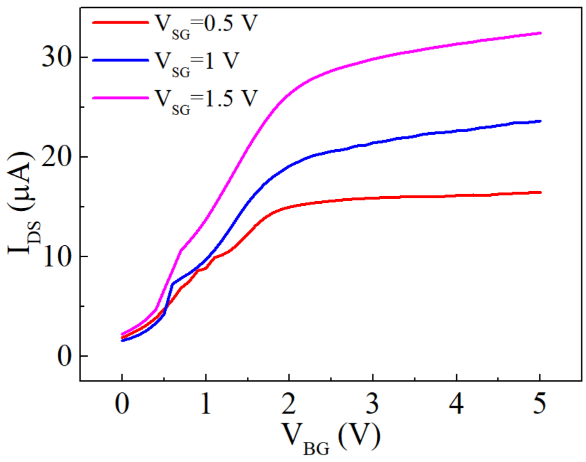

Figure S8. Effect of the side gate on the transfer curve of SMG-MOS for V_{DS} = 1.5 V.

Figure S9 aims to provide information about the stability of IGZO performance. In particular, considering the IGZO temperature dependence and the need of a device capable of maintaining constant properties regardless the ambient temperature, we have realized a protective cover with the aim to at least partially mitigate the temperature effect. In our new design the IGZO channel is protected by a cover while source and drain have maintained their original configuration. In this situation, we found a minor temperature dependence and thus less ambient dependence, as shown in **Figure S9**.

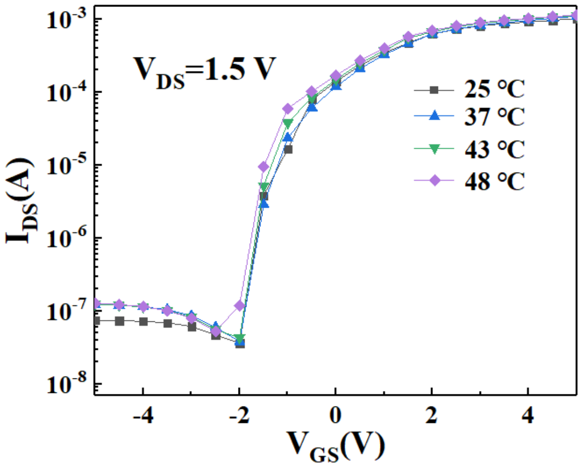

Figure S9. Temperature dependence for a SMG-MOS design when the IGZO channel cover was adopted.
